# Supplementary material for: LncRNA-LncDACH1 mediated phenotypic switching of smooth muscle cells during neointimal hyperplasia in male arteriovenous fistulas
Source: Nat Commun. 2024 May 3;15:3743. doi: 10.1038/s41467-024-48019-4 (PMC11068796; doi:10.1038/s41467-024-48019-4)
Supplement: Supplementary file 1 — Supplementary Information [file 41467_2024_48019_MOESM1_ESM.pdf]

Supplementary Fig. 1

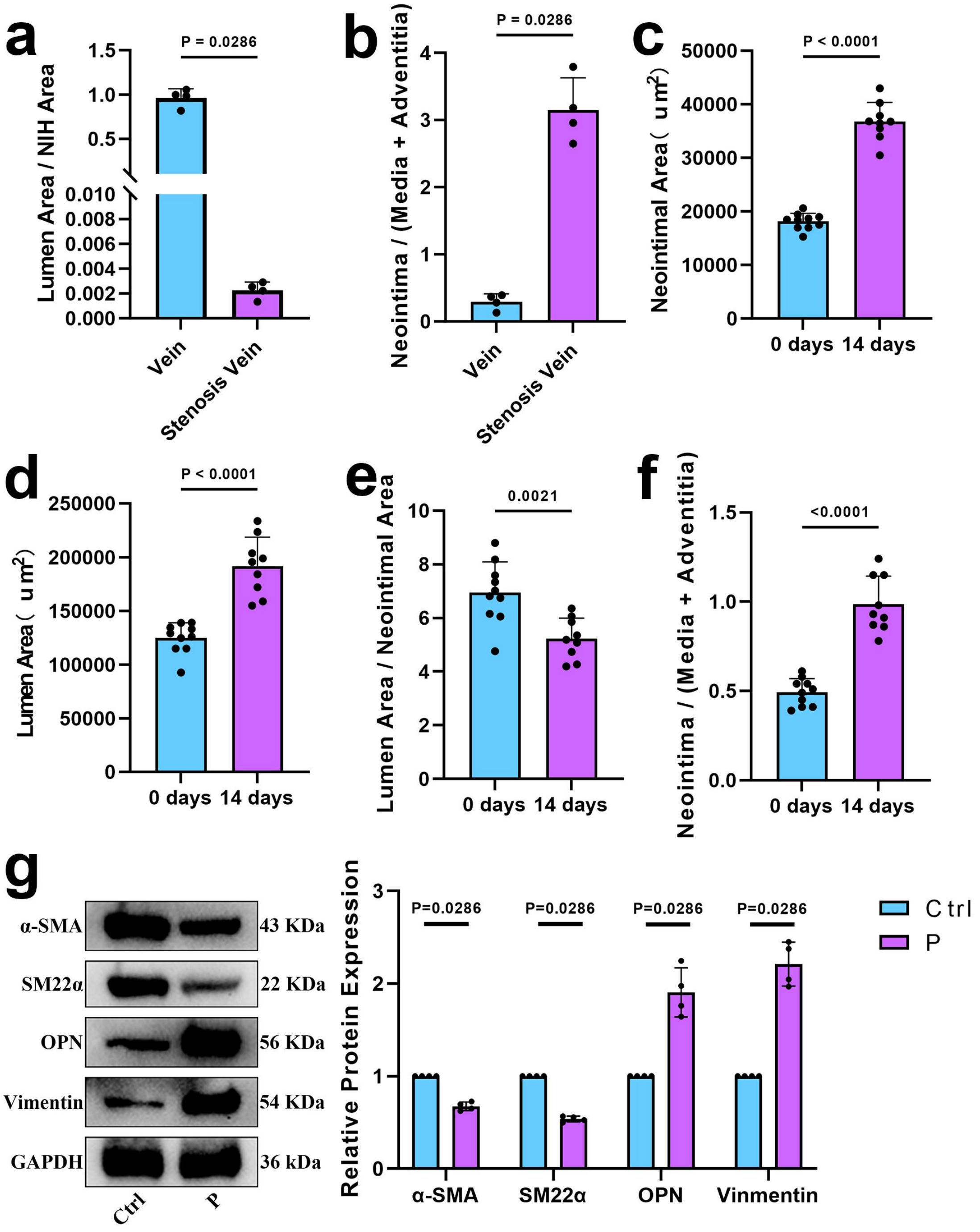

Supplementary Fig. 1 Morphometric analysis of human and animal samples. a-b Morphometric analysis of preoperative veins in human AVF (n=4) versus stenosis veins after AVF surgery (n=4) including Lumen Area / Neointimal Area ratio and Neointima / (Media + Adventitia) ratio. **c-f** Morphometric analysis of mouse before AVF (n=10) versus 14 days after

AVF (n=9) including Neointimal Area, Lumen Area, Lumen Area / Neointimal Area ratio and Neointima / (Media + Adventitia) ratio. **g** The expression level of VSMC differentiation phenotype markers and dedifferentiation phenotype marker protein after induction of human VSMCs by PDGF-BB (10ng/mL for 48h, n=4) were detected by Western Blot assay. The n numbers represent biologically independent samples. Data are presented as mean values +/- SD (a-g). P-values were determined by two-sided nonparametric tests (a-g). Source data are provided as a Source Data file.

Supplementary Fig. 2

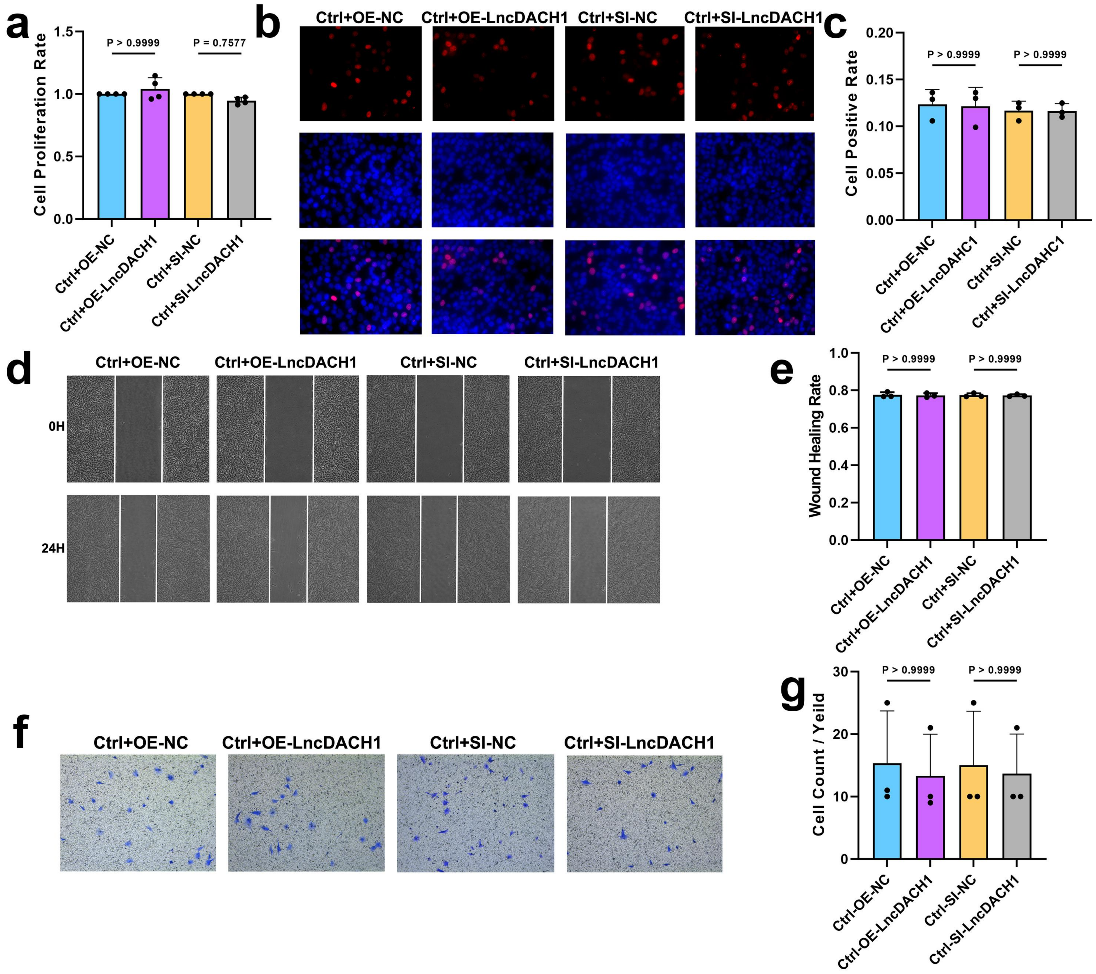

Supplementary Fig. 2 Modulation of LncDACH1 in differentiated VSMCs does not affect their proliferation, migration. a Effect of silencing or overexpression of LncDACH1 on the proliferation capacity of VSMC was examined by CCK-8 assay (n=4). **b, c** Effect of silencing or overexpression of LncDACH1 on VSMC proliferation capacity was examined by EdU staining assay (n=3). Scale bar, 25um. **d, e** Effect of silencing or overexpression of LncDACH1 on VSMC migration ability as measured by Wound Healing assay (n=3). Scale bar, 100um. **f, g** Effect of silencing or overexpression of LncDACH1 on VSMC migration ability was examined by Transwell assay (n=3). Scale bar, 50um. The n numbers represent biologically independent

samples. Data are presented as mean values +/- SD (a, c, e, g). P-values were determined by two-sided nonparametric tests (a, c, e, g). Source data are provided as a Source Data file.

Supplementary Fig. 3

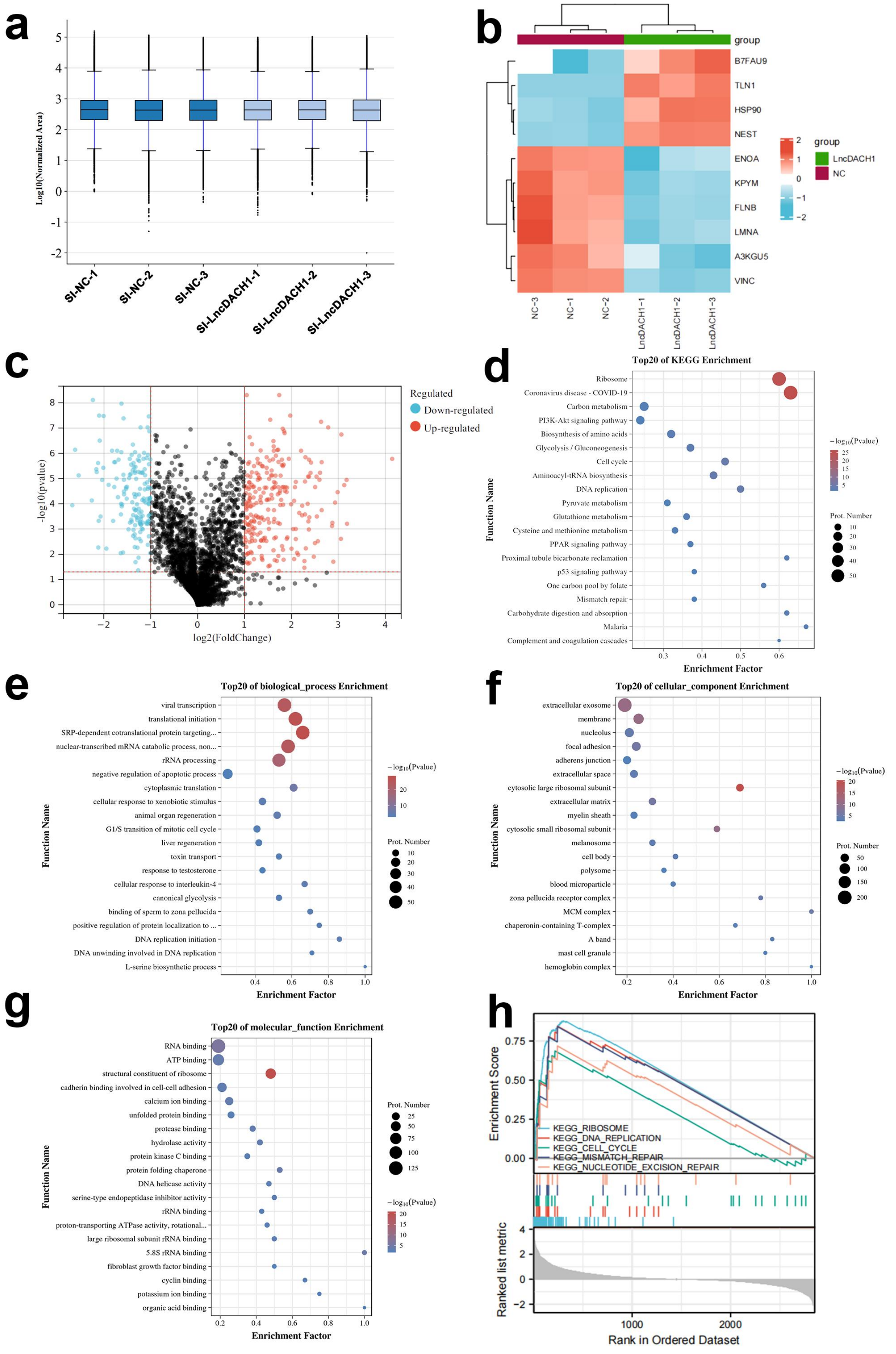

Supplementary Fig. 3 ITRAQ quantitative protein profiling bioinformatics analysis. a

Distribution of expression (abundance value) by sample of si-NC (n=3) and si-LncDACH1 (n=3) groups. **b-d** Bioinformatic analysis of the differential proteins of the si-NC (n=3) and si-LncDACH1 (n=3) groups in VSMC by iTRAQ quantitative protein profiling. **e-g** Biological Process (BP), Cell Component (CC) and Molecular Function (MF) analysis of differential genes. **h** GSEA enrichment analysis against HSP90. The n numbers represent biologically independent samples. P-values were determined by two-sided t-test (b, c) and one-sided hypergeometric test (d-g). P-values were adjusted for multiple testing (b, c, d-g). Boxes in all boxplots extend from the 25th to the 75th percentile and the lines indicate the median. The whiskers are drawn to the 5th and the 95th percentile. Source data are provided as a Source Data file.

Supplementary Fig. 4

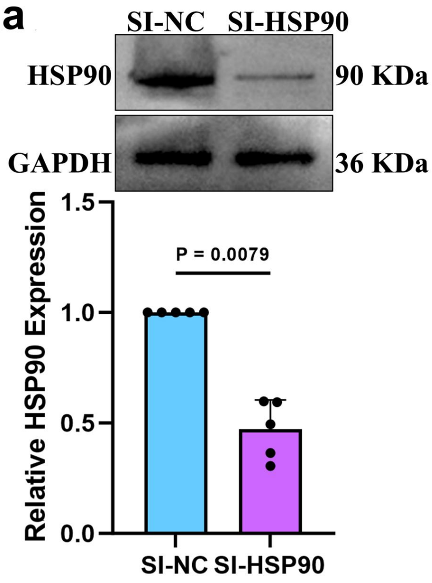

Supplementary Fig. 4 Silencing efficiency of HSP90. a Silencing efficiency of si-HSP90 in VSMC using Western Blot (n=5). The n numbers represent biologically independent samples. Data are presented as mean values +/- SD (a). P-values were determined by two-sided nonparametric tests (a). Source data are provided as a Source Data file.

Supplementary Fig. 5

a

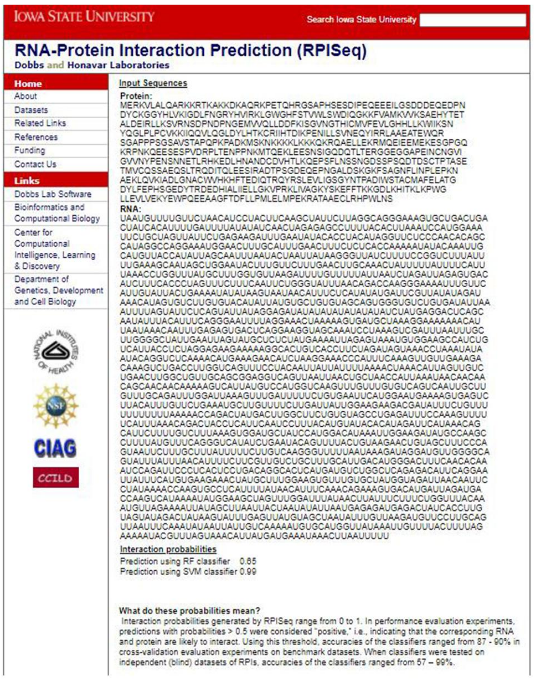

b

#	Protein region	RNA region	Interaction Propensity
1	101-152	814-897	129.73
2	98-149	814-897	117.09
3	223-274	814-897	114.06
4	273-324	814-897	111.50
5	276-327	814-897	109.92
6	323-374	814-897	105.29
7	151-202	814-897	103.23
8	373-424	814-897	101.81
9	26-77	814-897	99.33
10	423-474	814-897	99.25
11	426-477	814-897	96.76
12	176-227	814-897	96.39
13	126-177	814-897	94.88
14	548-599	814-897	93.78
15	551-602	814-897	92.83
16	376-427	814-897	92.60
17	148-199	814-897	92.32
18	326-377	814-897	92.14
19	226-277	814-897	90.72
20	123-174	814-897	87.97

c

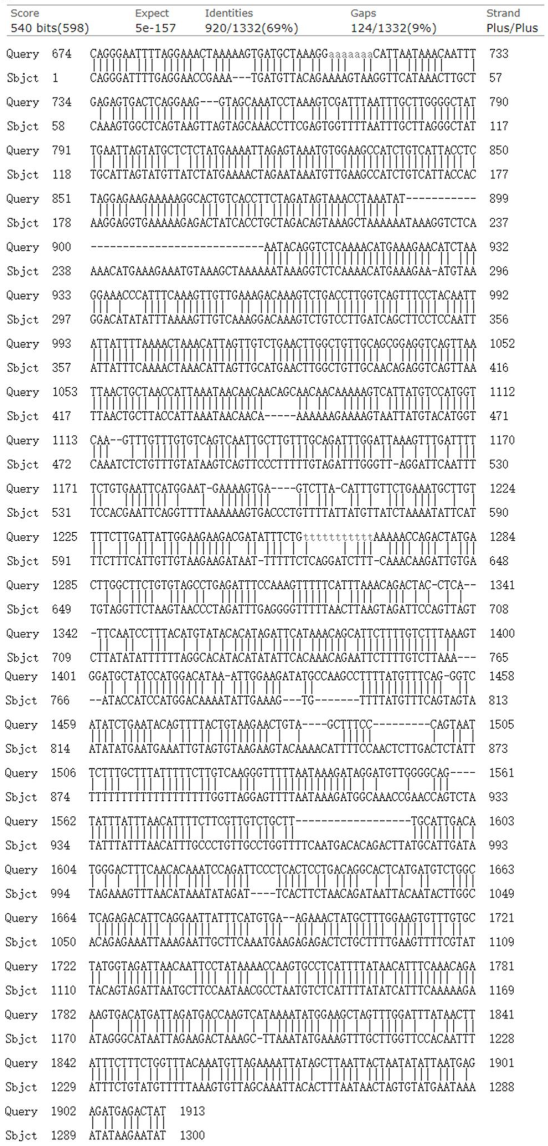

Supplementary Fig. 5 The binding sites between LncDACH1 and SRPK1. a Application of RPISeq database to predict the binding potential of LncDACH1 and SRPK1. **b** Application of the CatRapid database to predict the possible binding sites of LncDACH1 and SRPK1. **c** Comparison of the conservation of the mouse LncDACH1 (774-1251) fragment with the human homologous fragment.

Supplementary Fig. 6

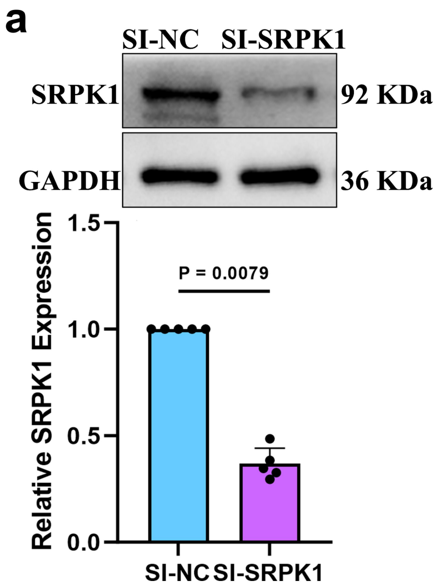

Supplementary Fig. 6 Silencing efficiency of SRPK1. a Silencing efficiency of si-SRPK1 in VSMC using Western Blot (n=5). The n numbers represent biologically independent samples. Data are presented as mean values +/- SD (a). P-values were determined by two-sided nonparametric tests (a). Source data are provided as a Source Data file.

Supplementary Fig. 7

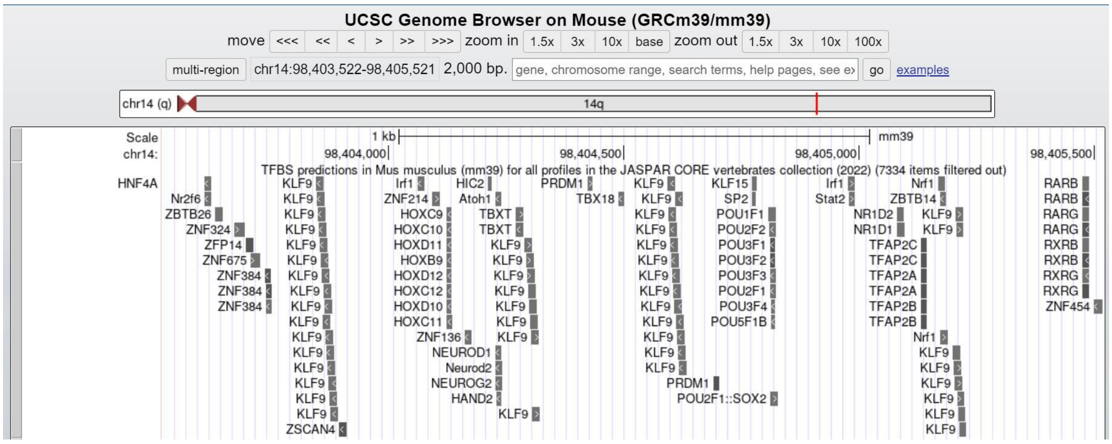

Supplementary Fig. 7 Transcription factors bound to the LncDACH1 promoter sequences. a Prediction of transcription factors binding to LncDACH1 promoter sequences using UCSC and JASPAR databases.

Supplementary Fig. 8

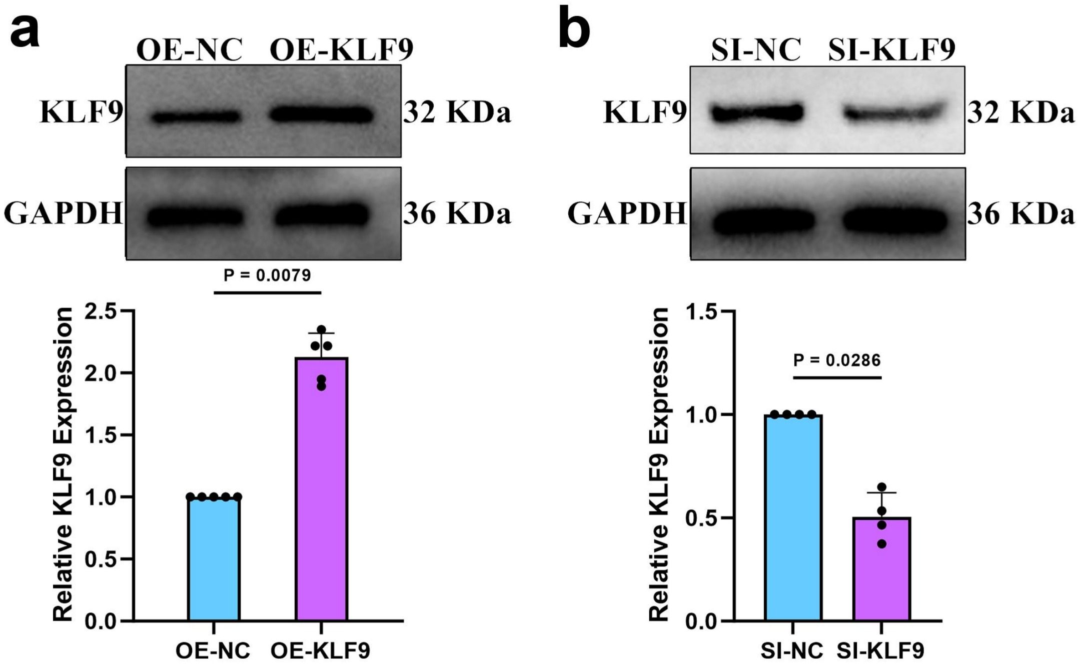

Supplementary Fig. 8 Transfection efficiency of KLF9. a Overexpression efficiency of pcDNA3.0-KLF9 in VSMC using Western Blot (n=5). **b** Silencing efficiency of si-KLF9 in VSMC using Western Blot (n=4). The n numbers represent biologically independent samples. Data are presented as mean values +/- SD (a, b). P-values were determined by two-sided nonparametric tests (a, b). Source data are provided as a Source Data file.

Supplementary Fig. 9

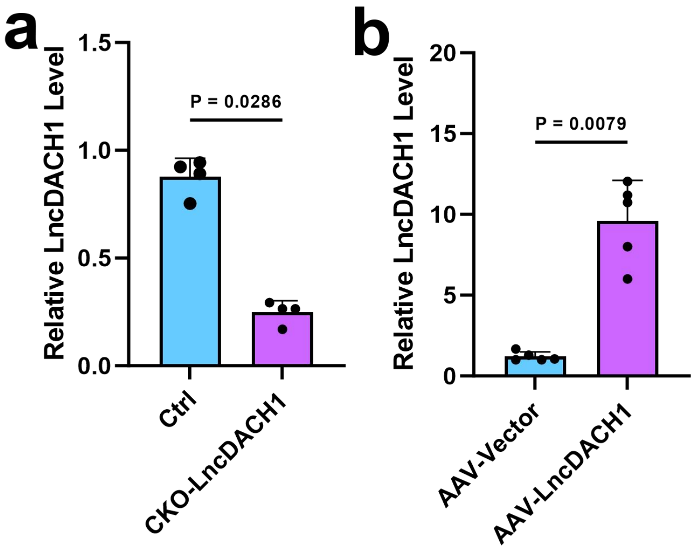

Supplementary Fig. 9 Expression level of LncDACH1 in CKO-LncDACH1 and AAV-LncDACH1 mice. a Expression levels of LncDACH1 in vascular tissues of LncDACH1 (flox+/flox, cre-) versus LncDACH1 (flox+/flox+, cre+) mice were measured by qRT-PCR (n=4 at each group). **b** Expression levels of LncDACH1 in vascular tissues of AAV-Vector and AAV-LncDACH1 mice were measured by qRT-PCR (n=5 at each group). The n numbers represent biologically independent samples. Data are presented as mean values +/- SD (a, b). P-values were determined by two-sided nonparametric tests (a, b). Source data are provided as a Source Data file.

Supplementary Fig. 10

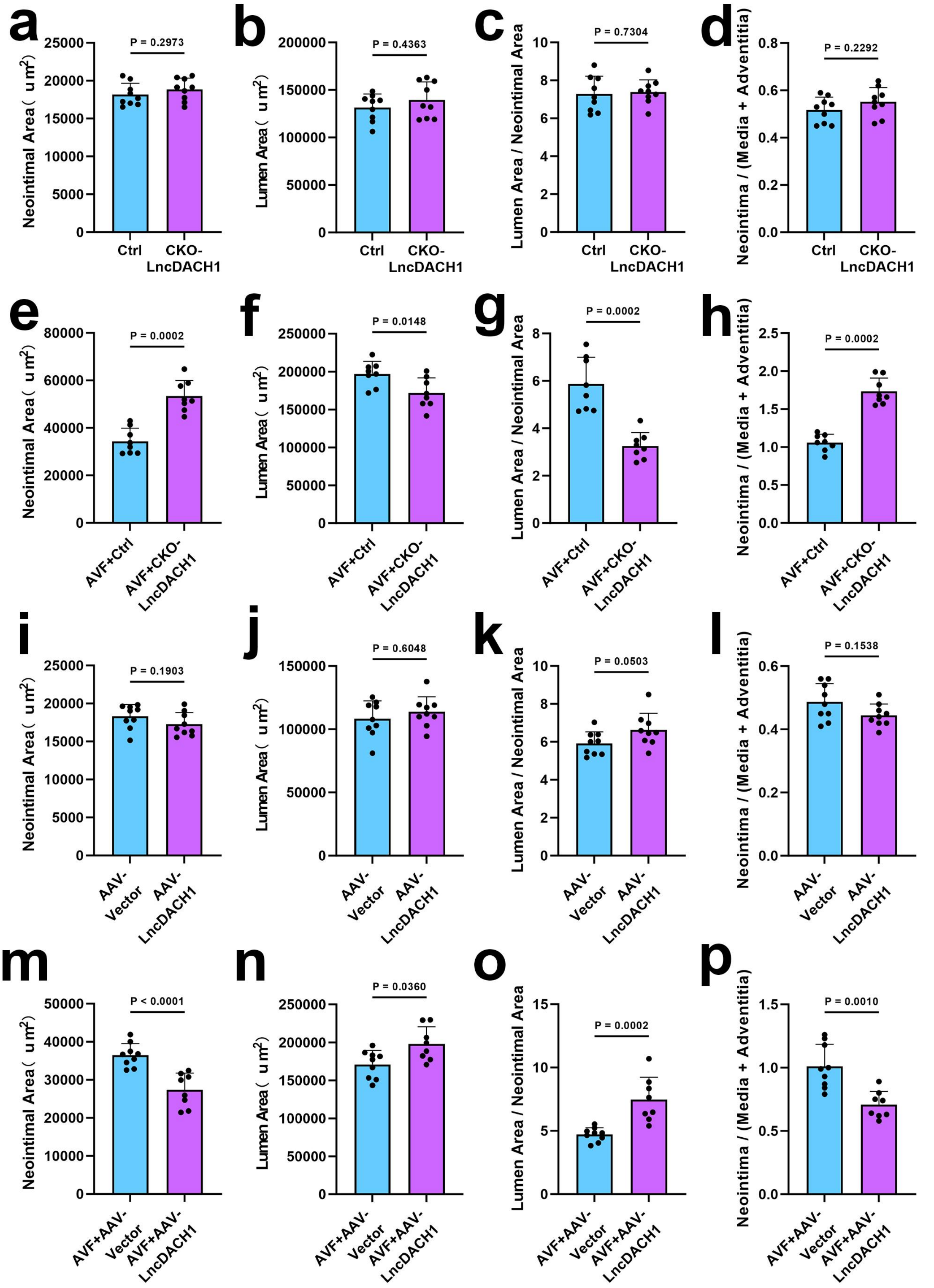

Supplementary Fig. 10 Morphometric analysis of CKO-LncDACH1 and AAV-LncDACH1 mouse samples. a-d Morphometric analysis of mouse without established AVF in CTRL (n=9)

and CKO-LncDACH1 (n=9) including Neointimal Area, Lumen Area, Lumen Area / Neointimal Area ratio and Neointima / (Media + Adventitia) ratio. **e-h** Morphometric analysis of mouse with established AVF in CTRL (n=8) and CKO-LncDACH1 (n=8) including Neointimal Area, Lumen Area, Lumen Area / Neointimal Area ratio and Neointima / (Media + Adventitia) ratio. **i-l** Morphometric analysis of mouse without established AVF in AAV-Vector (n=9) and AAV-LncDACH1 (n=9) including Neointimal Area, Lumen Area, Lumen Area / Neointimal Area ratio and Neointima / (Media + Adventitia) ratio. **m-p** Morphometric analysis of mouse with established AVF in AAV-Vector (n=9) and AAV-LncDACH1 (n=8) including Neointimal Area, Lumen Area, Lumen Area / Neointimal Area ratio and Neointima / (Media + Adventitia) ratio. The n numbers represent biologically independent samples. Data are presented as mean values +/- SD (a-p). P-values were determined by two-sided nonparametric tests (a-p). Source data are provided as a Source Data file.

Supplementary Fig. 11

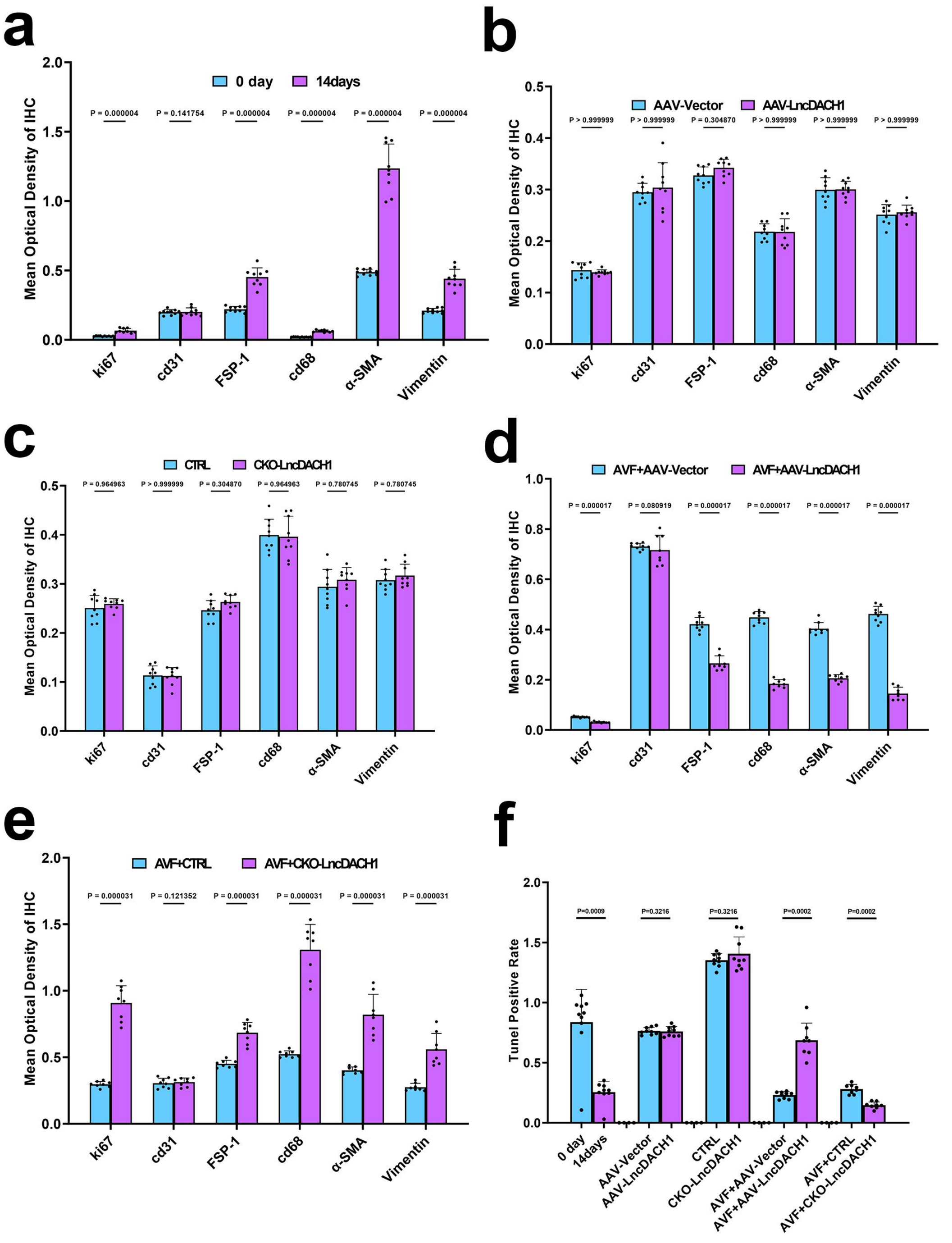

Supplementary Fig. 11 LncDACH1 affected NIH in the context of AVF by regulating VSMC phenotype switching (Statistical Analysis). a The expression level of Ki67, CD68, CD31, α-SMA, Vimentin and FSP-1 in mouse before AVF (n=10) versus 14 days after AVF (n=9) were detected by immunohistochemistry assay. **b** The expression level of Ki67, CD68,

CD31, α-SMA, Vimentin and FSP-1 in mouse without established AVF in AAV-Vector (n=9) and AAV-LncDACH1 (n=9) were detected by immunohistochemistry assay. **c** The expression level of Ki67, CD68, CD31, α-SMA, Vimentin and FSP-1 in mouse without established AVF in CTRL (n=9) and CKO-LncDACH1 (n=9) were detected by immunohistochemistry assay. **d** The expression level of Ki67, CD68, CD31, α-SMA, Vimentin and FSP-1 in mouse with established AVF in AAV-Vector (n=9) and AAV-LncDACH1 (n=8) were detected by immunohistochemistry assay. **e** The expression level of Ki67, CD68, CD31, α-SMA, Vimentin and FSP-1 in mouse with established AVF in CTRL (n=8) and CKO-LncDACH1 (n=8) were detected by immunohistochemistry assay. **f** The expression levels of Tunel in the above groups by immunofluorescence assay. The n numbers represent biologically independent samples. Data are presented as mean values +/- SD (a-f). P-values were determined by two-sided nonparametric tests (a-f). Source data are provided as a Source Data file.

Supplementary Fig. 12

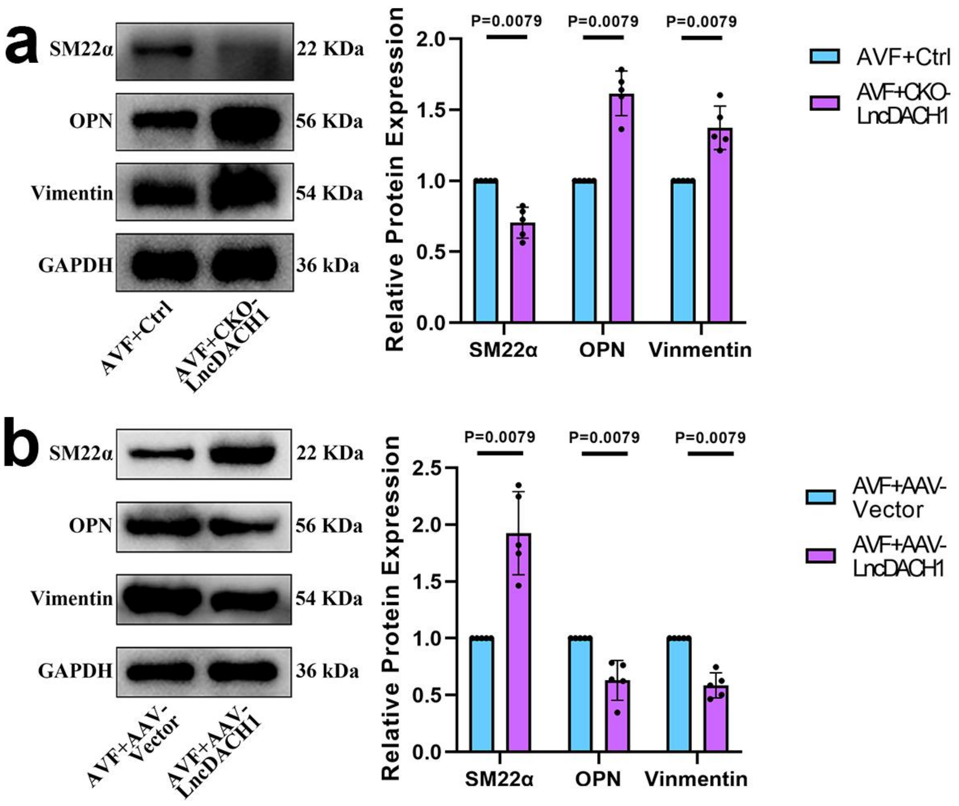

Supplementary Fig. 12 Effects on VSMC phenotype markers after establishment of AVF in mouse with CKO-LncDACH1 and AAV-LncDACH1. a The expression level of VSMC differentiation phenotype markers and dedifferentiation phenotype marker protein with established AVF in CTRL (n=5) and CKO-LncDACH1 (n=5) were detected by Western Blot assay. **b** The expression level of VSMC differentiation phenotype markers and dedifferentiation phenotype marker protein with established AVF in AAV-Vector (n=5) and AAV-LncDACH1 (n=5) were detected by Western Blot assay. The n numbers represent biologically independent samples. Data are presented as mean values +/- SD (a, b). P-values were determined by two-sided nonparametric tests (a, b). Source data are provided as a Source Data file.

Supplementary Tables

Supplementary Table 1. Characteristics of the participants in human samples.

Patient Characteristics	Vein (n=4)	Stenoisis Vein (n=4)	*P* Value
Age at time of surgery, years (median)	57	60	NS
Sex	4M;0F	4M;0F	NS
Active smoker (%)	0	0	NS
Hypertension (%)	75	66.7	NS
Diabetes (%)	50	75	NS
Coronary arterial disease (%)	33.3	50	NS
Venous thromboembolic disease (%)	0	0	NS

M, male; F, female; The n numbers represent biologically independent samples. Data are presented as mean values +/- SD. P-values were determined by two-sided nonparametric tests. NS, no significant (P >0.05) .

Supplementary Table 2. Sample size of AVF animal model.

Experimental group	Initial sample size	A	B	Final sample size
0 day	10	/	/	10
14 days (AVF)	10	1	0	9
AVF + CTRL	10	2	0	8
AVF + CKO-LncDACH1	10	1	1	8
AVF + AAV-Vector	10	0	1	9
AVF + AAV- LncDACH1	10	1	1	8

Exclusion reasons：**A** Surgery operation; **B** Death within 24 hours after surgery

Supplementary Table 3. si-RNA used for experiments

Mouse LncDACH1-sense	CACCCUAGUUUCUUUCAAUTT
Mouse LncDACH1-antisense	AUUGAAAGAAACUAGGGUGTT
Mouse HSP90-sense	GCAAGAACAUCGUCAAGAAGUTT
Mouse HSP90-antisense	UUCUUGACGAUGUUCUUGCGGTT
Mouse SRPK1-sense	AGAAGUUUGUAGCAAUGAAAGTT
Mouse SRPK1-antisense	UUCAUUGCUACAAACUUCUUUTT
Mouse KLF9-sense	GCUGUGGGAAAGUCUAUGGAATT
Mouse KLF9-antisense	UUCCAUAGACUUUCCCACAGCTT
Mouse Scramble-sense	UUCUCCGAACGUGUCACGUTT
Mouse Scramble-antisense	ACGUGACACGUUCGGAGAATT

Supplementary Table 4. PCR Primers used for experiments

Mouse LncDACH1-F	GTGACTCAGGAAGGTAGCAAATC
Mouse LncDACH1-R	CCTAGAGGTAATGACAGATGGC
Mouse LncDACH1(flox/flox)-F	CCAGAAGCACCCAGGACATTGTTGT
Mouse LncDACH1(flox/flox)-R	ACATCACAGAGCCACTGTA AGGAGTT
Mouse SMMHC-CreERT2-F1	TGACCCCATCTCTTCACTCC
Mouse SMMHC-CreERT2-R1	AGTCCCTCACATCCTCAGGTT
Mouse SMMHC-CreERT2-F2	CAGCCAACTTTACGCCTAGC
Mouse SMMHC-CreERT2-R2	TCTCAAGATGGACCTAATACGG
Mouse KLF9-F	GTGACCAAGGAACACGGTGACC
Mouse KLF9-R	CCTCATCGGGACTCTCCAGACTG
Mouse Matal1-F	GAGTTGTAGGCTTCTGTGTA
Mouse Matal1-R	AGGCTTGTGGTAGGTCAT
Mouse U6-F	GGAACGATACAGAGAAGATTAGC
Mouse U6-R	TGGAACGCTTCACGAATTTGCG
Mouse GAPDH-F	CAAGAAGGTGGTGAAGCAGG
Mouse GAPDH-R	CCACCCTGTTGCTGTAGCC
Mouse ChIP LncDACH1(P)-F	CACTTGGGAACTGGGAGAAA
Mouse ChIP LncDACH1(P)-R	AGTGTGCGTATGTGCATGTG
Mouse ChIP NC-F	AGCAACAGAAACACTGAAGCAA
Mouse ChIP NC-R	TGCCAAACTCTAGGCATTTCT
Human LncDACH1-F	TTGCAACAGCCAAGTTCATGC
Human LncDACH1-R	CTGTCCTTGATCAGCTTCCTCC
Human GAPDH-F	CAGGAGGCATTGCTGATGAT
Human GAPDH-R	GAAGGCTGGGGCTCATTT
