# Supplementary material for: LncRNA-LncDACH1 mediated phenotypic switching of smooth muscle cells during neointimal hyperplasia in male arteriovenous fistulas
Source: Nat Commun. 2024 May 3;15:3743. doi: 10.1038/s41467-024-48019-4 (PMC11068796; doi:10.1038/s41467-024-48019-4)
Supplement: Supplementary file 3 — Reporting Summary [file 41467_2024_48019_MOESM3_ESM.pdf]

nature portfolio

Corresponding author(s): Jundong Jiao

Last updated by author(s): Apr 15, 2024

Reporting Summary

Nature Portfolio wishes to improve the reproducibility of the work that we publish. This form provides structure for consistency and transparency in reporting. For further information on Nature Portfolio policies, see our Editorial Policies and the Editorial Policy Checklist.

Statistics

For all statistical analyses, confirm that the following items are present in the figure legend, table legend, main text, or Methods section.

n/a	Confirmed	
☐	☒	The exact sample size (*n*) for each experimental group/condition, given as a discrete number and unit of measurement
☐	☒	A statement on whether measurements were taken from distinct samples or whether the same sample was measured repeatedly
☐	☒	The statistical test(s) used AND whether they are one- or two-sided<br>*Only common tests should be described solely by name; describe more complex techniques in the Methods section.*
☒	☐	A description of all covariates tested
☐	☒	A description of any assumptions or corrections, such as tests of normality and adjustment for multiple comparisons
☐	☒	A full description of the statistical parameters including central tendency (e.g. means) or other basic estimates (e.g. regression coefficient) AND variation (e.g. standard deviation) or associated estimates of uncertainty (e.g. confidence intervals)
☐	☒	For null hypothesis testing, the test statistic (e.g. *F*, *t*, *r*) with confidence intervals, effect sizes, degrees of freedom and *P* value noted<br>*Give P values as exact values whenever suitable.*
☒	☐	For Bayesian analysis, information on the choice of priors and Markov chain Monte Carlo settings
☒	☐	For hierarchical and complex designs, identification of the appropriate level for tests and full reporting of outcomes
☐	☒	Estimates of effect sizes (e.g. Cohen's *d*, Pearson's *r*), indicating how they were calculated

Our web collection on statistics for biologists contains articles on many of the points above.

Software and code

Policy information about availability of computer code

Data collection	Absorbance as well as the chemiluminescence intensity were measured by Biotek SynergyHTX (USA). Data from the q-PCR experiments were collected using Roche LightCycler 480 II (Switzerland). Membranes were exposed using Bio Rad ChemiDoc MP Imaging System (USA). The related cells as well as the tissue images were acquired using Leica Microsystems DMI1 (Germany). Cell as well as tissue fluorescence images were acquired by Leica Microsystems DMIL (Germany).
Data analysis	Graph Pad Prism (Verison 9.3.0)<br>Image J (Verison 1.8.0)<br>PASS 15 (Verison 15.0.5)

For manuscripts utilizing custom algorithms or software that are central to the research but not yet described in published literature, software must be made available to editors and reviewers. We strongly encourage code deposition in a community repository (e.g. GitHub). See the Nature Portfolio guidelines for submitting code & software for further information.

Data

Policy information about availability of data

All manuscripts must include a data availability statement. This statement should provide the following information, where applicable:

- Accession codes, unique identifiers, or web links for publicly available datasets
- A description of any restrictions on data availability
- For clinical datasets or third party data, please ensure that the statement adheres to our policy

The mass spectrometry proteomics data generated in this study have been deposited in the ProteomeXchange Consortium via the iProX partner repository with the dataset identifier database[https://www.iprox.cn//page/project.html?id=IPX0005596000]. The full-length LncDACH1 Promoter sequence and the predicted binding sites of KLF9 were obtained through the UCSC database[https://genome.ucsc.edu/] and JASPAR database[https://jaspar.elixir.no/]. The predicted binding and sequence of LncDACH1 with SRPK1 was obtained through the RPISeq database[http://pridb.gdcb.iastate.edu/RPISeq/] and catRAPID database[http://s.tartaglialab.com/page/catrapid_group]. Source data are provided with this paper.

Research involving human participants, their data, or biological material

Policy information about studies with human participants or human data. See also policy information about sex, gender (identity/presentation), and sexual orientation and race, ethnicity and racism.

Reporting on sex and gender	In this experiment, we only collected samples from male patients to exclude the effect of estrogen on the results of this experiment (sex of participants was determined based on their sex at birth).
Reporting on race, ethnicity, or other socially relevant groupings	The samples are all from Chinese Han population.
Population characteristics	Preoperative AVF veins were collected from uraemic patients who were about to undergo AVF surgery, whereas stenosis AVF veins were collected from veins that were discarded during revision or reconstruction of focal stenosis surgery for AVF treatment. Only male sample was selected for this study with an age range of 52-72 years.
Recruitment	All participants in this study were enrolled in the Second Hospital of Harbin Medical University. The inclusion criteria for this study were 1. male patients with uremia 2. complete clinical data; 3. availability of specimens.<br>Exclusion criteria included 1. age less than 18 years; 2. postoperative AVF stenosis cause other than neointimal hyperplasia 3. combination of other serious vascular related diseases. Informed written consent was obtained from all participants.
Ethics oversight	The study were approved by the Ethics Committee of the Second Affiliated Hospital of Harbin Medical University. We informed all related patients of the use of these specimens and got the written informed consent.

Note that full information on the approval of the study protocol must also be provided in the manuscript.

Field-specific reporting

Please select the one below that is the best fit for your research. If you are not sure, read the appropriate sections before making your selection.

☒ Life sciences ☐ Behavioural & social sciences ☐ Ecological, evolutionary & environmental sciences

For a reference copy of the document with all sections, see nature.com/documents/nr-reporting-summary-flat.pdf

Life sciences study design

All studies must disclose on these points even when the disclosure is negative.

Sample size	We used power analysis to determine the animal sample size for the final experiment. we performed formal power analysis separately in vivo experiments by PASS 15 software.When performing power analysis, we assumed Alpha = 0.05 as well as Target Power > 0.90. For other experiments, sample size was based on the feasibility of the experiment, the availability of samples, and the certainty of results needed to obtain definitive results.
Data exclusions	No data was excluded.
Replication	In order to verify the reproducibility of the experiments in this study, we performed at least three replicate trials between different batches of samples for each group of experiments. The results showed that the experiments involved in this study were reproducible.
Randomization	All samples were randomly allocated into experimental groups.
Blinding	For animal study, the investigators were blinded for the animal allocation to the experimental groups. All data collection and analyses were performed in a blind manner. For in vitro study, blinding was not applicable because the investigator in charge of an experiment was responsible for cell culture, treatment, sample collection, and data analysis.

Reporting for specific materials, systems and methods

We require information from authors about some types of materials, experimental systems and methods used in many studies. Here, indicate whether each material, system or method listed is relevant to your study. If you are not sure if a list item applies to your research, read the appropriate section before selecting a response.

Materials & experimental systems

n/a	Involved in the study	
☐	☒	Antibodies
☐	☒	Eukaryotic cell lines
☒	☐	Palaeontology and archaeology
☐	☒	Animals and other organisms
☒	☐	Clinical data
☒	☐	Dual use research of concern
☒	☐	Plants

Methods

n/a	Involved in the study	
☒	☐	ChIP-seq
☒	☐	Flow cytometry
☒	☐	MRI-based neuroimaging

Antibodies

Antibodies used

Anti-HSP90 (mouse monoclonal, clone F-8); Santa Cruz; Cat # sc-13119; Lot# B2322; 1:1000 for WB 1:200 for IF

Anti-KLF9 (mouse monoclonal, clone A-5); Santa Cruz; Cat # sc-376422; Lot# I3020; 1:200 for WB

Anti-SRPK1 (rabbit polyclonal); Proteintech□Cat # 14073-1-AP; Lot# 00041414; 1:1000 for WB 1:50 for IF 1:50 for IP

Anti-GAPDH (mouse monoclonal); Proteintech□Cat # 60004-1-Ig; Lot# 10028231; 1:100000 for WB

Anti-Lamin B1 (mouse monoclonal); Proteintech□Cat # 66095-1-Ig; Lot# 10020247; 1:20000 for WB

Anti-AKT (rabbit monoclonal,clone 11E7); Cell Signaling Technology□Cat # 4685; Lot# 6; 1:1000 for WB

Anti-p-AKT (rabbit monoclonal,clone D9E)(Ser473); Cell Signaling Technology□Cat # 4060; Lot# 27; 1:2000 for WB

Anti-FLAG (rabbit monoclonal,clone D6W5B); Cell Signaling Technology□Cat # 14793; Lot# 7; 1:50 for IP

Anti-TAGLN (rabbit polyclonal); Abcam; Cat # ab14106; Lot# GR3380763-2; 1:1000 for WB

Anti-Opn (rabbit multiclonal , clone RM1018); Abcam; Cat # ab283656; Lot# 1002432-6; 1:1000 for WB

Anti-Vimentin (rabbit monoclonal , clone EPR3776); Abcam; Cat # ab92547; Lot# GR3258719-33; 1:1000 for WB

Anti-α-SMA (rabbit monoclonal , clone EPR5368); Abcam; Cat # ab124964 ; Lot# GR303485-23; 1:1000 for WB

Validation

Anti-HSP90 (mouse monoclonal) ; Suitable for: WB, IP, IF, IHC(P), FCM , ELISA ; https://www.scbt.com/p/hsp-90alpha-beta-antibody-f-8?requestFrom=search

Anti-Opn (rabbit multiclonal); Suitable for: IHC-P, WB, IP; https://www.abcam.cn/products/primary-antibodies/osteopontin-antibody-rm1018-ab283656.html

Anti-Vimentin (rabbit monoclonal); Suitable for: Flow Cyt (Intra), ICC/IF, WB, IHC-P, mIHC; https://www.abcam.cn/products/primary-antibodies/vimentin-antibody-epr3776-cytoskeleton-marker-ab92547.html

Anti-α-SMA (rabbit monoclonal)?Suitable for: WB, IHC-P, ICC/IF, Flow Cyt (Intra); https://www.abcam.cn/products/primary-antibodies/alpha-smooth-muscle-actin-antibody-epr5368-ab124964.html

Anti-KLF9 (mouse monoclonal) ; Suitable for: WB, IF, IHC(P), FCM , ELISA ; https://www.scbt.com/p/bteb1-antibody-a-5?requestFrom=search

Anti-SRPK1 (rabbit polyclonal)□Suitable for: FC, IF, IHC, IP, WB, ELISA□ https://www.ptgcn.com/products/SRPK1-Antibody-14073-1-AP.htm

Anti-GAPDH (mouse monoclonal)□Suitable for:FC, IF, IP, WB, ELISA□https://www.ptgcn.com/products/GAPDH-Antibody-60004-1-Ig.htm

Anti-Lamin B1 (mouse monoclonal); Suitable for:FC, IF, IHC, IP, WB, ELISA; https://www.ptgcn.com/products/LMNB1-Antibody-66095-1-Ig.htm

Anti-AKT (rabbit monoclonal);Suitable for:WB, IP, IHC-P, IF-IC, FC-FP; https://www.cellsignal.cn/products/primary-antibodies/akt-pan-11e7-rabbit-mab/4685

Anti-p-AKT (rabbit monoclonal,clone D9E)(Ser473); Suitable for:WB, W-S, IP, IHC-P, IF-IC, FC-FP; https://www.cellsignal.cn/products/primary-antibodies/phospho-akt-ser473-d9e-xp-rabbit-mab/4060?_=1684301087361&Ntt=4060&tahead=true

Anti-FLAG (rabbit monoclonal); Suitable for: WB, IP, IHC-P, IF-IC, FC-FP, ChIP; https://www.cellsignal.cn/products/primary-antibodies/dykddddk-tag-d6w5b-rabbit-mab-binds-to-same-epitope-as-sigma-s-anti-flag-m2-antibody/14793?site-search-type=Products&N=4294956287&Ntt=14793&fromPage=plp&_requestid=401338

Anti-TAGLN (rabbit polyclonal); Suitable for: ICC/IF, WB; https://www.abcam.cn/products/primary-antibodies/taglntransgelin-antibody-ab14106.html

Eukaryotic cell lines

Policy information about cell lines and Sex and Gender in Research

Cell line source(s)	The HEK-293T cell line (Otwobiotech Technology, HTX1559) and mouse (Otwobiotech Technology, HTX1886) and human VSMC cell lines (Otwobiotech Technology, HTX2352) were purchased from Otwobiotech Technology (China).
Authentication	The HEK-293T, Mouse-VSMC and Human-VSMC cell line was obtained from suppliers. Cell authentication is based on their morphology, growth conditions and specific gene expression.
Mycoplasma contamination	Cells lines used in this study tested negative for mycoplasma contamination.
Commonly misidentified lines (See ICLAC register)	No commonly misidentified cell lines were used in this study.

Animals and other research organisms

Policy information about studies involving animals; ARRIVE guidelines recommended for reporting animal research, and Sex and Gender in Research

Laboratory animals	Wildtype C57BL/6J mice at the age of 6 to 8 weeks (Animal Center of the Second Affiliated Hospital of Harbin Medical University, Harbin, China) were used. SMMHC-CreERT2 mice at the age of 6 to 8 weeks (Cyagen Biosciences Inc , USA) were used. LncDACH1 (flox+/flox+, Cre-) mice and LncDACH1 (flox+/flox+, Cre-) mice (Biocytogen Co, China) at the age of 6 to 8 weeks were used. They were fed in the standard room without pathogens, and the light cycle (12 h light to 12 h dark), humidity (50% ±5%) and temperature (20°C to 22°C) were specifically controlled.
Wild animals	Wild animals were not involved in this experiment.
Reporting on sex	In this experiment, we applied only samples from male mice to exclude the effect of estrogen on the results of this experiment.
Field-collected samples	The study did not involved the use of samples collected from the flied.
Ethics oversight	All experiments were conducted in accordance with protocols approved by the Ethics Committee on Use and Care of Animal Center of Harbin Medical University. All mice were bred according to the protocol of the Instituional Animal Care and Use Committee (IACUC).

Note that full information on the approval of the study protocol must also be provided in the manuscript.

Plants

Seed stocks	*Report on the source of all seed stocks or other plant material used. If applicable, state the seed stock centre and catalogue number. If plant specimens were collected from the field, describe the collection location, date and sampling procedures.*
Novel plant genotypes	*Describe the methods by which all novel plant genotypes were produced. This includes those generated by transgenic approaches, gene editing, chemical/radiation-based mutagenesis and hybridization. For transgenic lines, describe the transformation method, the number of independent lines analyzed and the generation upon which experiments were performed. For gene-edited lines, describe the editor used, the endogenous sequence targeted for editing, the targeting guide RNA sequence (if applicable) and how the editor was applied.*
Authentication	*Describe any authentication procedures for each seed stock used or novel genotype generated. Describe any experiments used to assess the effect of a mutation and, where applicable, how potential secondary effects (e.g. second site T-DNA insertions, mosiacism, off-target gene editing) were examined.*
